# Viral suppression rate among children tested for HIV viral load at the Amhara Public Health Institute, Bahir Dar, Ethiopia

**DOI:** 10.1186/s12879-019-4058-4

**Published:** 2019-05-14

**Authors:** Melashu Balew Shiferaw, Demeke Endalamaw, Mulat Hussien, Manamnot Agegne, Desalegn Amare, Fikirte Estifanos, Dinbere Temesgen

**Affiliations:** 1 Amhara Public Health Institute, Bahir Dar, Ethiopia; 2 College of Medicine and Health Sciences, Bahir Dar University, Bahir Dar, Ethiopia

**Keywords:** HIV, Children, Viral load suppression, ART, Ethiopia

## Abstract

**Background:**

Human immunodeficiency virus (HIV) infected children represent a very vulnerable population for anti-retroviral therapy (ART) drug resistance. As a global target, 90% of patients receiving ART should have HIV-RNA viral suppression. A threshold of *>* 1000 RNA copies/ml is used to define non-suppressed viral load. If it is confirmed in the laboratory, adherence should be addressed and should be followed by the switch to second-line ART. Therefore, the aim of this study was to assess the rate of viral load suppression among children tested at the Amhara Public Health Institute (APHI), Bahir Dar.

**Methods:**

Institutional based cross-sectional study design was conducted from July 01, 2017 to June 30, 2018, in children under the age of 15 years. Socio-demographic, clinical and HIV1RNA viral load data were collected from the excel database. The data were analyzed in SPSS 20.0 statistical software.

**Results:**

A total of 1567 children, age ranged from one to 14 years, were tested for HIV viral load. Of which, about 54% were males. Children were treated using nevirapine-based (76.7%), efavirenz-based (21.8%) and protease inhibitor-based (1.5%) anti-retroviral drugs. Non-suppressed HIV viral load was found in 28.3% of the participants. High viral load (> 1000 cp/ml) were found in 24% of the children below the age of five years. Children on nevirapine-based treatment had about two times more non-suppressed viral load (Adjusted odds ratio [AOR]: 1.90; 95%CI: 1.41–2.56; *P* < 0.001) compared to those who had efavirenz-based treatment. However, adherence (P: 0.204) was not associated with non-suppressed viral load.

**Conclusions:**

There was a high rate of non-suppressed HIV viral load among children tested at APHI. Specifically, the odds of having a non-suppressed viral load was higher in NVP based treatment users. Hence, comprehensive management and follow up of children on ART, and testing for resistance as well as viral load could help to reduce the problem in advance.

## Background

Children infected with human immunodeficiency virus (HIV) have exceptionally higher morbidity and mortality compared to adults. As children acquire HIV when the immune system is immature, it results in high rates of viral replication, high viral load, high rates of CD4 destruction, accumulation of mutations in the viral population and faster rates of disease progression [1]. They are also susceptible to opportunistic infections, including tuberculosis and other bacterial infections that are associated with high rates of mortality [2].

Globally, there were about 1.8 million HIV-infected children; more than 80% live in sub-Saharan Africa [3]. In the absence of any intervention, up to 52 and 75% of children die before the age of two and five years respectively [2]. According to the 2017 World Health Organization (WHO) report, Ethiopia is one of the highly affected countries with an estimated 62,000 children living with HIV. The estimated number of deaths of children due to HIV was 2900. The reported number of children receiving anti-retroviral therapy (ART) was 21,700 [4].

The Joint United Nations Program on HIV/AIDS (UNAIDS) has set the three 90s target for 2020. Meaning that 90% of people living with HIV know their status, 90% of those diagnosed receive ART, and 90% receiving ART should have HIV-RNA viral suppression [5]. Good adherence to ART and retention in care are necessary tasks to achieve viral suppression, prevent viral failure, diminish viral transmission and reduce HIV/AIDS-related deaths [6].

Some children may take ART, but these drugs may not control the HIV replication [7]. Poorly controlled HIV can be due to many factors, including lack of health care, poor medication adherence, drug resistance and drug toxicity [7]. According to WHO, children represent a very vulnerable population: one out of two children newly diagnosed with HIV is infected with virus harboring resistance to efavirenz (EFV) and nevirapine (NVP), thus at high risk of suboptimal treatment [8].

Treatment failure can be clinical failure, immunologic failure, viral failure or any combination of the three. The goal of therapy following treatment failure is to achieve and maintain viral suppression as measured by a plasma viral load below the limits of detection using the most sensitive assay [7].

Viral load is the gold standard for HIV treatment monitoring that could show the amount of HIV genetic material (RNA) circulating in the blood plasma. The WHO established viral suppression of 90% in patients on ART to have an undetectable HIV viral load (VL) as a global target [5]. Achieving viral suppression protects the body’s immune system, helps people living with HIV stay healthy and prevents transmission of HIV to other people. If a patient on treatment is found to have detectable viral replication, it may be due to poor drug adherence and/or drug resistance [9]. In Ethiopia, the three 90s target has been introduced by the ministry of health [2]. Many viral load testing sites have been expanded to implement the third 90.

HIV-infected children are considered a priority group for routine viral load monitoring [10]. A threshold of *>* 1000 RNA copies/ml is used to define viral failure [2]. If the viral failure is confirmed in the laboratory, adherence should be addressed well, and then followed by a switch to second-line ART [11]. Thus, regular monitoring and evaluation about viral suppression is very important to achieve the established targets and take necessary corrective actions. However, there is limited data regarding viral suppression among children in Ethiopia including our setting. Therefore, the aim of this study was to assess the status of viral load suppression rate among children attending the APHI viral load testing site.

## Methods

### Study design and study area

A cross sectional study was conducted to determine the magnitude of viral suppression rates among children attending HIV viral load testing in APHI, Bahir Dar, Ethiopia. The study was carried out from July 01, 2017 to June 30, 2018. In the Amhara region alone, there are five viral load testing sites. This allows the implementation of routine or need-based viral load testing to monitor viral suppression. The APHI is one of the five viral load testing centers to serve HIV infected patients who came from 81 peripheral health facilities in the Amhara region. The institute also provides the service for neighboring regions such as Benishangul-Gumuz. The laboratory provides only viral load testing service, no HIV clinic in the institute, through sample referral system. Samples collected at different health facilities were transported to the APHI through postal and/or vehicle that were dedicated to the specimen transport. All specimens were transported and submitted to the APHI central reception, and after laboratory analysis results were sent back to the health facilities through postal services. According to the 2017 regional health bureau report, the prevalence of HIV in Amhara region was 1.6%, and there were more than 33,041 HIV infected patients tested for HIV viral load including children under the age of 15 years.

### Study population

All children who got HIV viral load testing service in the APHI during the study period were our study population. Each child sample was considered as a study unit. Children with complete requisition and plasma samples were used as inclusion criteria. Requests with missing age and poor specimen quality such as haemolysis were excluded from the study. All samples that fit the inclusion criteria were included in the study and selected consecutively.

### Data collection procedure

The data extraction tool was prepared by the investigators and used to collect data. Variables of interest were collected from the requests and excel database.

When postal couriers and/or vehicle drivers submitted samples at the central reception of the APHI, specimen quality and completeness of requests were checked. Socio-demographic and clinical data were extracted from the laboratory request using the abstraction tool.

Each child specimen was coded using bar code and submitted to the virology reference laboratory for viral load testing. Trained laboratory analysts investigated the viral load test following the standard operating procedure. It was done using advanced molecular techniques. In brief, nucleic acid extraction was done from the children’s plasma samples using an automated m2000sp machine (Abbott Molecular inc., Germany). Extraction reagents were used as follows: magnetic particle to trap RNA; M lysis to destruct membrane; mwash1 and mwash2 to purify the nucleic acid; and elusion buffer to break the bond between magnetic particle and the RNA. The unrelated RNA sequence was also introduced into each specimen at the beginning of sample preparation as an internal control to demonstrate that the process proceeded correctly for each sample. Negative, low positive and high positive controls were used in each test order to evaluate run validity. Then, the extracted nucleic acid was mixed with amplification reagents (thermo stable polymerase enzyme in buffered solution, oligonucleotides, quencher, reference dyes and activation reagent) to amplify and detect HIV1 RNA using an automated m2000rt machine (Abbott Molecular inc., USA). After laboratory analysis, results were interpreted as not detected and detected (number of HIV virus RNA copies). The lower and higher detection limit of the machine is 150 and 10,000,000 RNA copies per ml, respectively. Results with greater than 1000 HIV virus RNA copies per ml was considered as not suppressed viral load results.

### Statistical analysis

The data in excel format were exported to the SPSS version 20.0 statistical packages for analysis. Frequencies were presented to describe study participants. The association between outcome (viral suppression) and independent (age, sex, test reason, treatment combination and adherence) variables were measured using backward likelihood ratio (LR) method in the multivariable logistic regressions. Significant association between study variables and interpretation of data were done using the adjusted odds ratio (AOR) and 95% confidence interval. *P* value < 0.05 was considered as significant association.

## Results

### Socio-demographic characteristics

A total of 1567 children under the age of 15 years were tested for HIV viral load during the study period. More than half, 975 (62.1%), were 10 years and above; range: 1–14 years. Under-five children accounted about 5% of the total participants. The majority of the participants (1467 [93.6%]) were from the Amhara region. Male children were about 54% of the total study participants (Table 1).

**Table 1:** Characteristics of HIV infected children referred for viral load testing at APHI, Bahir Dar, from 01 July 2017 to 30June 2018

Characteristics	Responses	Number	%
Age	< 3 years	45	2.9
	3–9 years	550	35.1
	+ 10 years	972	62.0
Under-five	No	1493	95.3
	Yes	74	4.7
Sex	Female	727	46.4
	Male	840	53.6
Region	Amhara	1467	93.6
	Benishangul-Gumuz	100	6.4

### Clinical profile and viral suppression status

About 95% of the children had WHO stage one category. There was a good HIV treatment adherence in 1515 (96.7%) participants. Viral load testing was requested to confirm anti-retroviral drug failure in 247 (15.8%) suspects, and to monitor routine viral load in 1320 (84.2%) HIV infected children. The children were treated using NVP based (76.7%), EFV based (21.8%) and PI based (1.5%) anti-retroviral drugs. The most widely used drug combinations were 3TC+ NVP+ AZT (1117 [71.3%]) and 3TC+ EFV+ AZT (239 [15.3%]).

Of the total 1567 children tested, the viral suppression rate was only 71.7%. In other words, 28.3% (444/1567) of the participants had non-suppressed HIV viral load. Among suspected children, 86 (34.8%) had non-suppressed viral load. Children referred for routine viral load testing had 27.1% viral failures (viral load results greater than 1000 copies/ml). The high viral copy among under-five children was 24.3% (18/74) (Table 2).

**Table 2:** Clinical profile of HIV infected children referred for viral load testing to the APHI, Bahir Dar, from 01 July 2017 to 30June 2018

Variables	Category	Number	%
WHO stage	I	1483	94.6
	II	59	3.8
	III	19	1.2
	IV	6	0.4
Adherence	Good	1515	96.7
	Fair	31	2.0
	Poor	21	1.3
Test reason	Suspect	247	15.8
	Routine	1320	84.2
Treatment	EFV based	342	21.8
	NVP based	1202	76.7
	PI based	23	1.5
Viral load	Not detected	1053	67.2
	Detected	514	32.8
Viral suppression	Suppressed	1123	71.7
	Not suppressed	444	28.3

Abbreviations: *EFV* efavirenz, *NVP* nevirapine, *PI* protease inhibitor

### Associated factors for viral suppression failure

In multiple logistic regressions, being suspected of anti-retroviral drug failure and NVP based treatment were significantly associated with the viral suppression failure. The suspects had significantly higher prevalence of viral suppression failure compared to the prevalence among children diagnosed for routine viral load (AOR: 1.38; 95%CI: 1.03–1.84; P: 0.032). Those children who had NVP based treatment was about two times viral suppression failure (AOR: 1.90; 95%CI: 1.41–2.56; *P* < 0.001) compared to those children who had EFV based treatment (Table 3). However, adherence (*P* = 0.204), age (*P* = 0.421) and sex (*P* = 0.085) were not associated with non-suppressed viral load.

**Table 3:** Associated factors of viral suppression failure among children referred for HIV viral load testing at APHI, Bahir Dar, from 01 July 2017 to 30June 2018

Variable	Viral suppression		Crude OR (95%CI)	Adjusted OR (95%CI)	*P*-value
	Suppressed	Not suppressed			
Age					
< 3 years	16	8	1.36 (0.58–3.22)	–	–
3–9 years	396	175	1.204 (0.96–1.51)	–	–
+ 10 years	711	261	1		
Sex					
Male	617	223	1	1	
Female	506	221	1.21 (0.97–1.51)	1.22 (0.97–1.52)	0.085
Test reason					
Routine	962	358	1	1	
Suspect	161	86	1.44 (1.08–1.92)	1.38 (1.03–1.84)	0.032
Treatment					
EFV based	278	64	1	1	
NVP based	829	373	1.95 (1.45–2.63)	1.90 (1.41–2.56)	< 0.001
PI based	16	7	1.90 (0.75–4.81)	1.81 (0.71–4.60)	0.212
Adherence					
Good	1087	428	1		
Fair	24	7	0.74 (0.32–1.73)	–	–
Poor	12	9	1.905 (0.80–4.55)	–	–

Hosmer and Lemeshow Test**:** X^2^ = 4.001, and P value = 0.676

Abbreviations: *CI* confidence interval, *EFV* efavirenz, *NVP* nevirapine, *PI* protease inhibitor, *OR* odds ratio

## Discussion

In this study, we evaluated the rate of viral load suppression failure among children tested at APHI because early identification of such problems is essential to make interventions. The overall rate of non-suppressed HIV viral load was 28%. This shows the suppression rate is still at 72%, which is very far from the 90% target to be achieved in 2020 [2, 5]. In line with this finding, 25.4% of HIV infected children had viral failure in Tanzania [12]. Surprisingly, very elevated viral failure occurred in 66% of children in Malawi [13] and 43.1% of children in Kenya [14]. Such non-suppressed HIV viral load could indicate how much the virus is available to damage the system. The CD4 count goes down and the immune system weakens. In addition, the risk of HIV transmission is greater as patients with high HIV viral loads are likely to be more infectious [15, 16].

The WHO target of 90% could be achieved even in developing countries. A study done in Uganda showed that the magnitude of the non-suppressed viral load was 11% among HIV infected children [17], which is about three times less compared to the suppression rate found in our study. This highlights the need to closely follow and properly manage children on ART to suppress the viral load more in our setting. The emergency operation center currently established in the Amhara region may help to improve problems regarding high HIV viral load.

In this study, children enrolled under NVP based treatment had significantly higher (31%) viral suppression failure (AOR: 1.90; 95% CI: 1.41–2.56; *P* < 0.001) compared to those children who had EFV based treatment. Similarly, 26% NVP resistance was found in Malawi [13]. A significant rise in viral load may be a sign that the virus is replicating even in the presence of HIV drugs. This means patients become resistant to at least one drug in the combination. Resistance to an anti-HIV drug is a problem because it means that the patients can no longer use that drug to keep their viral load low. Poor adherence and mutation can lead to drug resistance [16]. Although our study didn’t include sequencing of the genetic material, some studies revealed that NVP had a high rate of resistance to HIV among infants [18, 19].

Adherence to ART is one of the important factors of treatment outcome. Poor adherence may result in a non-suppressed viral load, leading to opportunistic infections, drug resistance and ultimately mortality [20]. This is strongly supported by different studies across the world. For instance, studies done in Thailand, Vietnam and Uganda showed strong associations between non-suppressed viral load and poor adherence [18, 21, 22]. However, adherence was not associated with non-suppressed viral load in our study. Similarly, a study conducted in Uganda also showed low HIV viral suppression rates following the intensive adherence counseling [23]. The result of good adherence, together with non-suppressed viral load might highlight the presence of drug resistance among children on ART. Hence, programs should start testing for resistance in addition to viral load monitoring.

## Conclusions

There was a high rate of non-suppressed HIV viral load (28%) among children tested at APHI. NVP based treatment and being suspected of anti-retroviral failure was significantly associated with the viral load suppression failure. Hence, comprehensive management and follow up of children on ART, and the programs start testing for resistance as well as viral load could help to reduce the problem in advance.

## Abbreviations

3TC: Lamivudine
AOR: Adjusted Odds Ratio
APHI: Amhara Public Health Institute
ART: Anti-retroviral Therapy
AZT: Zidovudine
EFV: Efavirenz
HIV: Human Immunodeficiency Virus
LR: Likelihood Ratio
NVP: Nevirapine
PI: Protease Inhibitor
UNAIDS: United Nations Program on HIV/AIDS
WHO: World Health Organization report
